# Cutaneous Metastasis of Carcinoma Buccal Mucosa: A Rare Presentation

**DOI:** 10.7759/cureus.25812

**Published:** 2022-06-10

**Authors:** Aaditya Prakash, Amitabh Upadhyay

**Affiliations:** 1 Radiation Oncology, Tata Main Hospital, Jamshedpur, IND; 2 Oncology, Tata Main Hospital, Jamshedpur, IND

**Keywords:** cancer immunotherapy, radiation therapy, skin metastasis, carcinoma oral cavity, cutaneous metastasis

## Abstract

Cutaneous metastasis (CM) of head-and-neck squamous cell carcinoma is rarely reported. Here, we report a case of a 78-year-old male who had already had a surgically treated case of carcinoma of the right buccal mucosa one and a half months back but presented with a near local site recurrence and subsequently developed distant skin metastases to the lower neck and upper trunk during treatment. Local site recurrence was confirmed with a biopsy, but benign-looking lesions in the lower neck and upper trunk were developed during the last week of treatment, which later on kept growing in size and after the biopsy came positive for malignancy. Although there was a complete response at the recurred site of malignancy, there was a progression of disease at the initially benign-looking lesion in the lower neck and upper trunk, which later on was proven to be cutaneous malignancy on histopathology. This case has cemented the fact that the chance of the presence of occult skin metastasis at the time of diagnosis in primary or recurrent malignancies. Thus, tissue biopsy should be done with a high index of suspicion for cutaneous malignancy associated with head and neck cancers.

## Introduction

Oral cavity carcinoma accounts for 30% of all head-and-neck cancers [1]. It is more common in Southeast Asia because of the widespread use of tobacco (in any form), betel nuts, etc., especially in India [2]. The common sites of distant metastasis are the lung, liver, and bones [3]. However, cutaneous metastasis (CM) of squamous cell carcinomas of the head and neck is rare. CM generally develops near the primary site of recurrence, then spreads outwards. Here, we report a case who had already been treated for carcinoma of the right side buccal mucosa but presented with local site recurrence as primary and subsequently developed distant skin metastases to the lower neck and upper trunk during the treatment of the primary recurrence site.

## Case presentation

**Figure 1:**
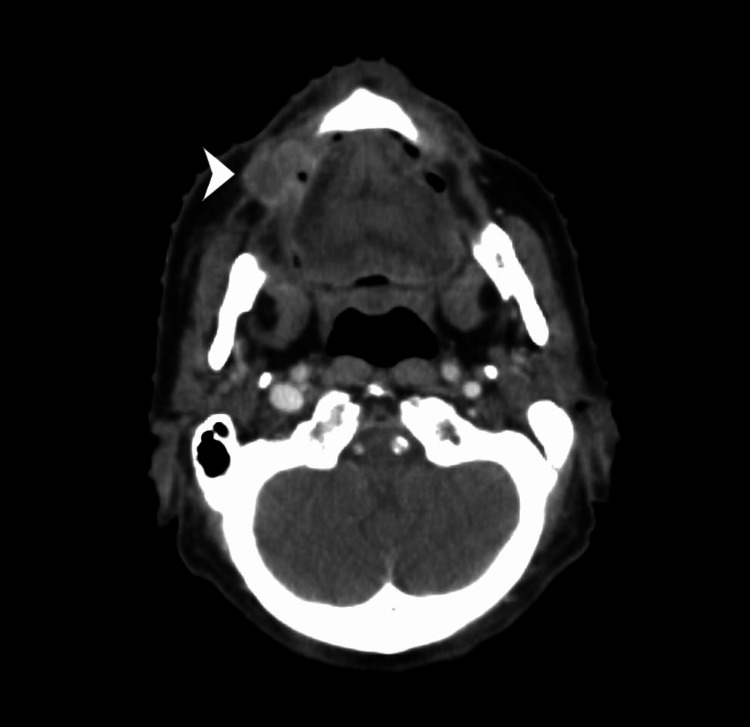
Whole body PET CT image with CT section of tumour (white arrow head is showing the tumour).

**Figure 2:**
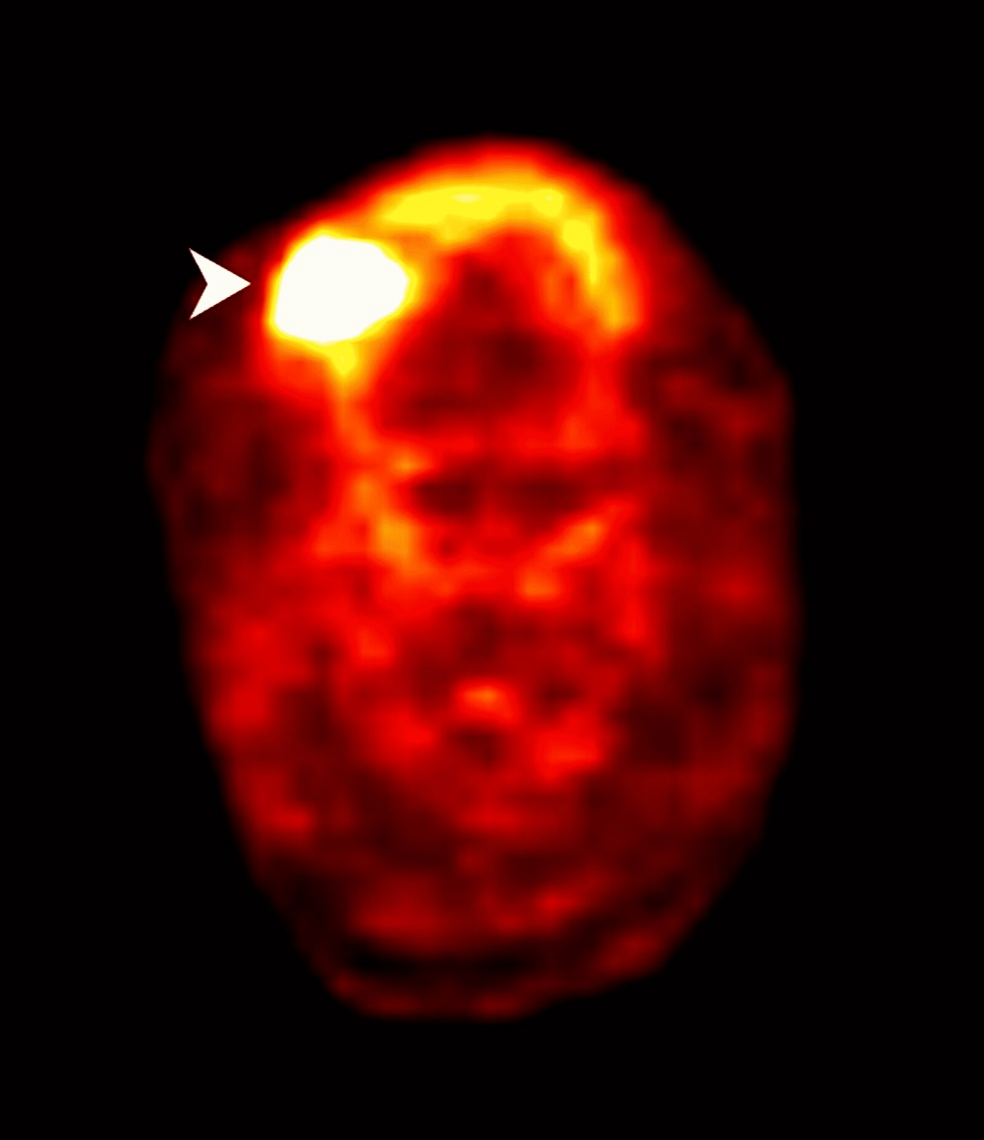
Whole Body PET CT image with PET section of tumour (white arrow head is showing the tumour).

A 78-year-old male, with comorbidities of ischemic heart disease (IHD), was a known case of invasive squamous cell carcinoma of the right side buccal mucosa for which he underwent wide local excision of the lesion with right supra-omohyoid neck dissection of nodes at another private centre. His pathological staging was pT1N0M0 (stage I). He was kept on follow-up. After one and a half months, he developed a non-healing lesion near the previously post-operated site. He was referred to our centre for further work up and management. The lesion was slightly anterior to the previously operated site. The biopsy from the recurred lesion was reported as a moderately differentiated squamous cell carcinoma, which confirmed the findings of local recurrence. Whole-body PET-CT was suggestive of localized disease with a 17 mm × 15 mm × 19 mm lesion involving the right buccal mucosa (Figures 1-2). There was no evidence of any disease elsewhere in the body. Re-surgery was ruled out in view of the poor left ventricular ejection fraction (LVEF) of 34%. The patient was scheduled for local site radiation therapy after Institutional Board approval.

He was planned for a definitive dose of external beam radiotherapy. The planning target volume (PTV) received 66 Gy in 30 fractions with the image-guided intensity-modulated radiotherapy (IG-IMRT) technique on Varian TrueBeam by 6 MV photons. His PTV included the local recurrence site and ipsilateral nodal coverage. Concurrent weekly chemotherapy was deferred in view of comorbidities and general Condition.

**Figure 3:**
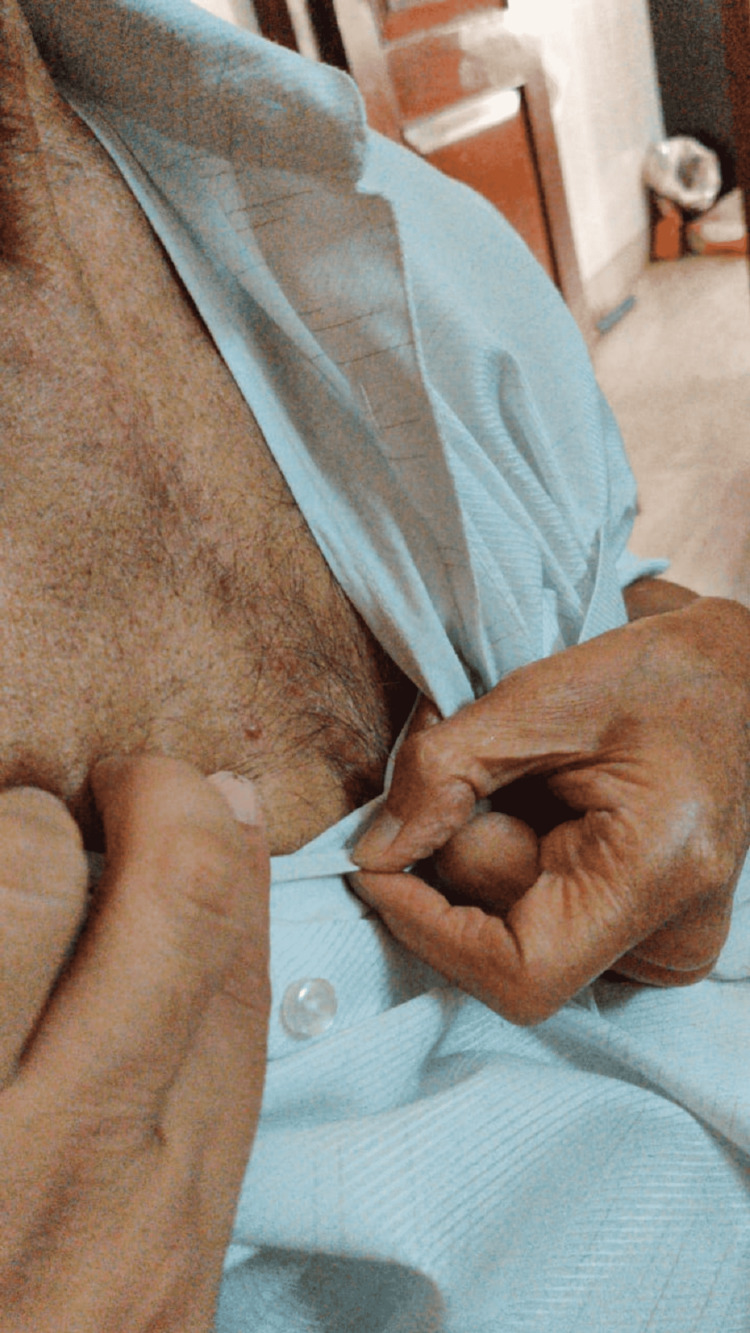
Erythematous nodules over chest wall.

**Figure 4:**
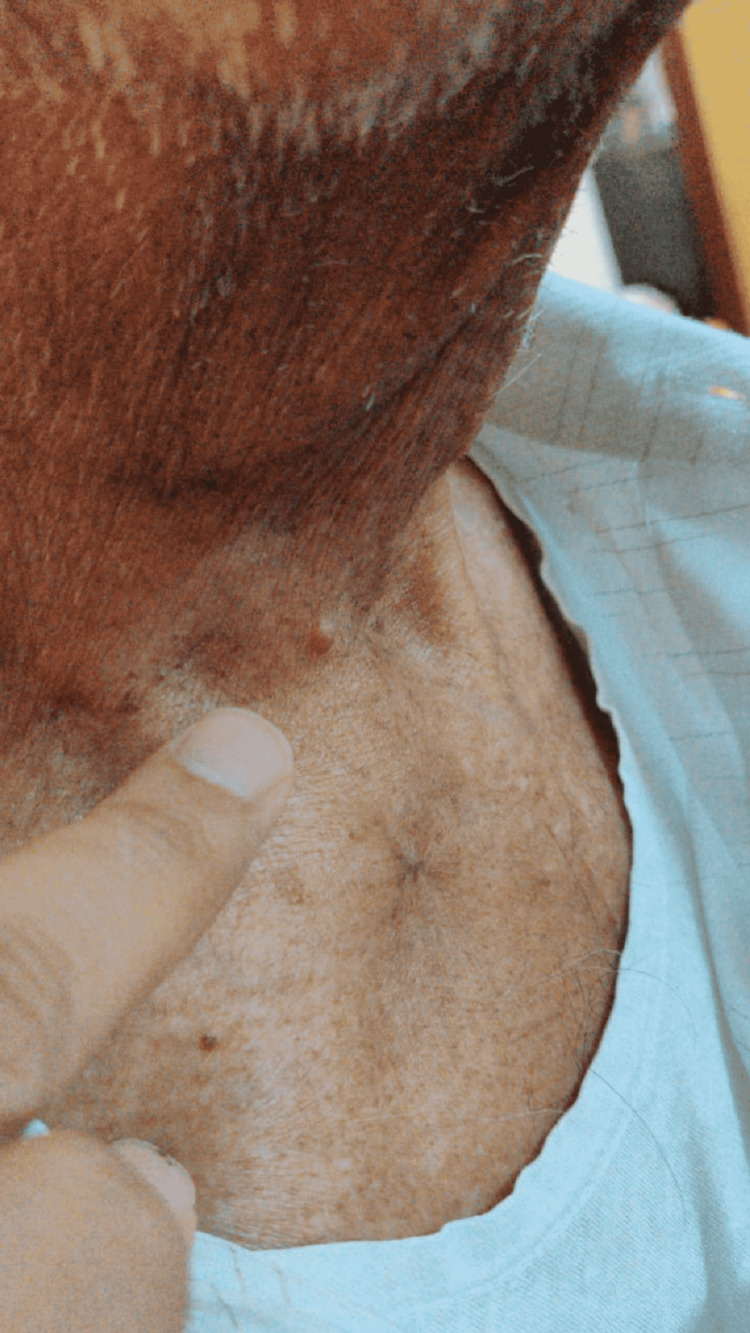
Erythematous nodules over neck.

He developed erythematous sub-centimetric nodules over the right lower neck and upper chest wall in the last week of radiation therapy (Figures 3-4). He was kept under observation in view of his benign-looking appearance. After a few days of radiation therapy completion, the number and size of lesions increased with pus discharge from some of the lesions. His pus was sent for culture and sensitivity, which is positive for *Staphylococcus aureus* infection.

**Figure 5:**
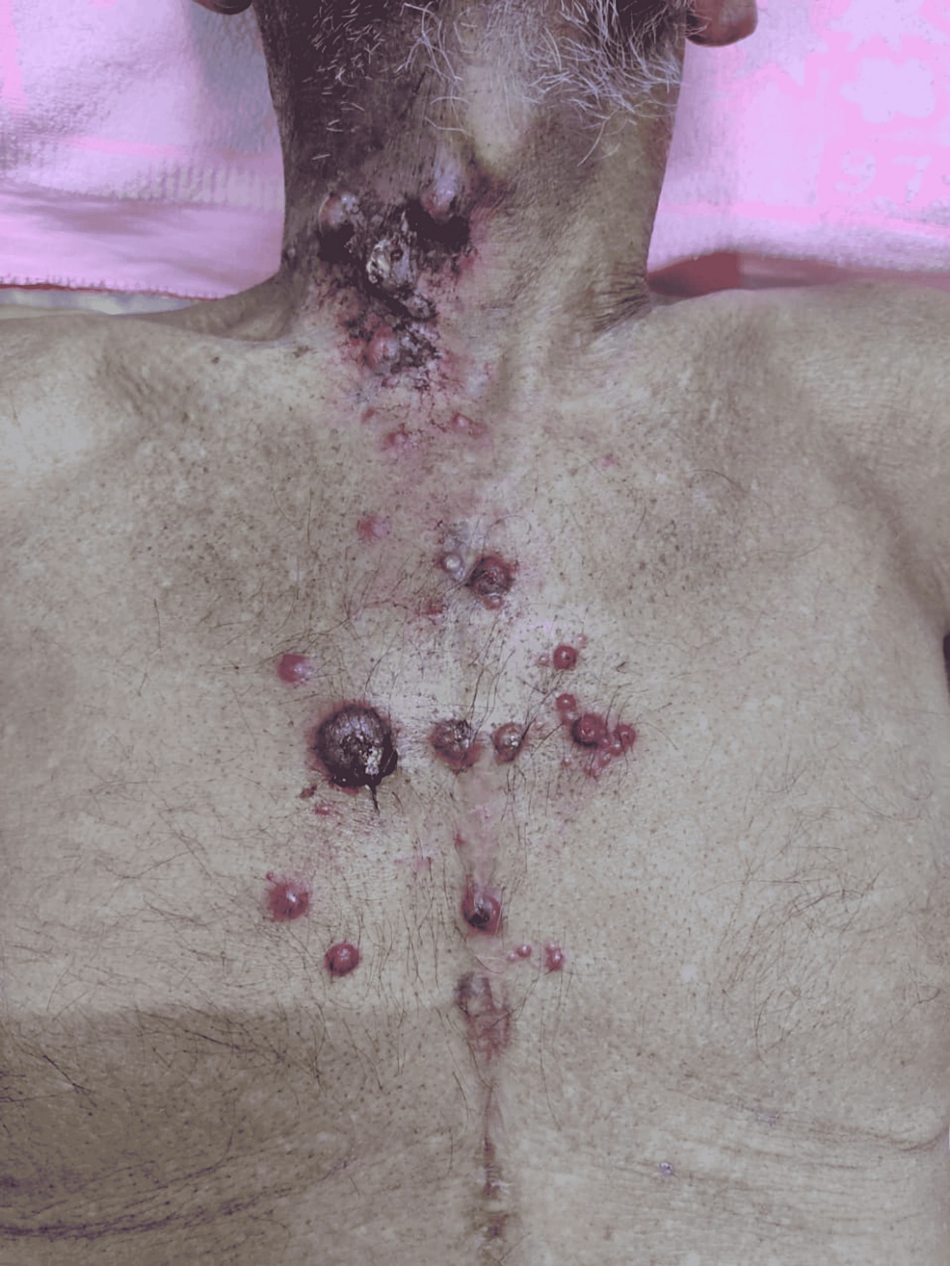
Biopsy taken from chest wall lesions.

**Figure 6:**
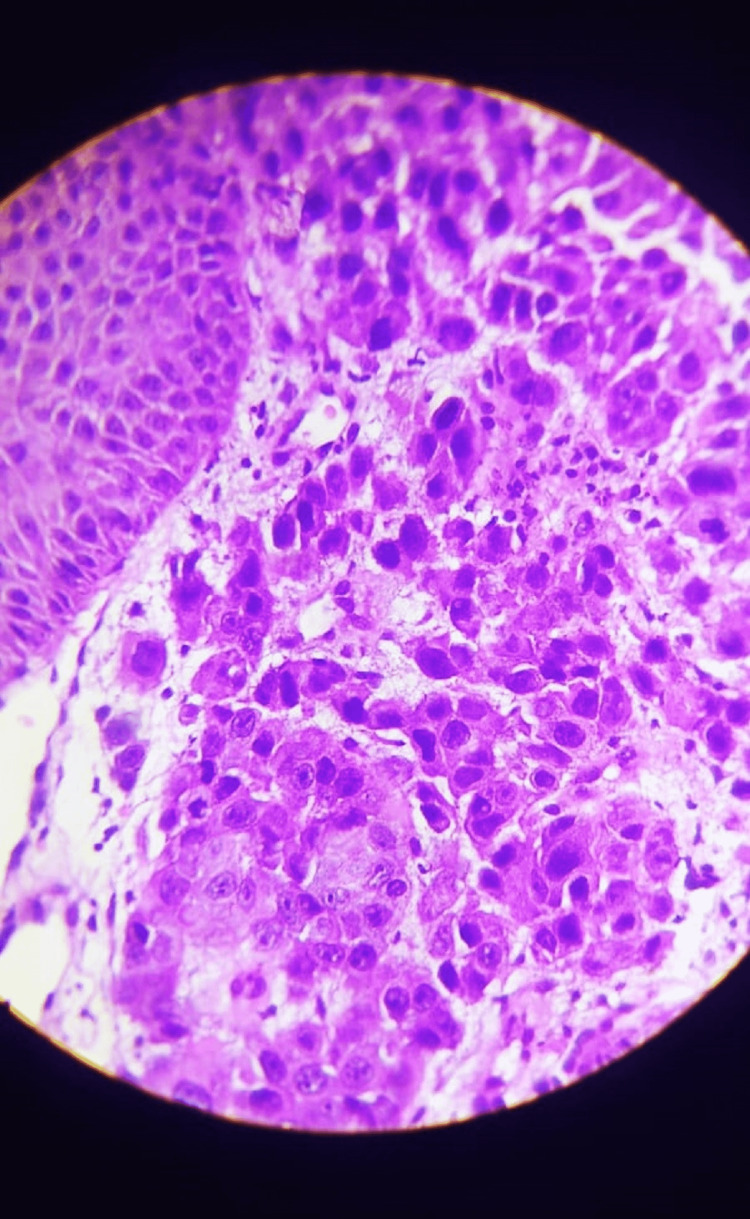
H and E slide showing keratinization, nuclear pleomorphism, and cellular dissociation which is suggestive of squamous cell carcinoma (40× with cellular details of biopsy taken from chest wall lesions).

He was kept on antibiotics, which was sensitive to *S. aureus* infection, as per the report. But the lesion kept on increasing in size, so a biopsy was taken from the local site, which came as metastatic squamous cell carcinoma (Figures 5-6).

Immunotherapy was recommended but deferred after consulting with family members about the patient's poor prognosis and general condition. After three months of treatment, the patient succumbed due to disease progression.

## Discussion

The incidence of cutaneous metastasis from most malignancies varies between 0.7% and 9% [4]. The most common primary malignancies associated with cutaneous metastasis are breast cancer and lung cancer. Cutaneous metastases are distinguishable from primary cutaneous squamous cell carcinomas because they are completely separated from the overlying epidermis.

There are some case reports of head and neck malignancies with cutaneous metastasis that showed variable presentations.

Schultz and Schwartz reported a rare presentation of cutaneous metastasis from hypopharyngeal malignancy [5]. Veraldi et al. reported a few cases of cutaneous metastasis from laryngeal cancers [6]. These case reports also include those cases that presented upfront with cutaneous metastasis in primary settings. Srinivasan et al. reported a case with carcinoma of the left buccal mucosa with distant cutaneous metastases to the right-side chest wall, post-treatment at four months [1]. In general, distant metastases of head and neck squamous cell carcinoma (HNSCC) spread to other sites, and among them, the most common sites of distant metastases are usually the lung, liver, and bone.

Possible mechanisms for cutaneous metastasis as suggested by Kmucha and Troxel are direct spread, local spread, or distant spread [7]. These modes of metastasis are not based on experimental evidence but are hypothetical. Cutaneous metastasis indicates an overall poor prognosis. In their study, Berger and Fletcher reported that the length of survival was approximately three months after skin metastasis in HNSCC [8,9]. Verma et al. reported a case of chest wall skin metastasis from carcinoma on the left side of the tongue, post-treatment 48 months [10]. This case report showed that cutaneous metastasis can be presented as the first sign of spread without going on to appear as a local recurrence or secondary to other organs.

Overall, in such cases, treatment remains palliative in intent. Individual case-by-case treatment in the form of surgery, chemotherapy, immunotherapy, radiation therapy, or a combination of these may be used. Although surgery shows some extent of benefit in terms of survival in such cases, unfortunately, whatever treatment (s) are administered, cutaneous metastasis shows poor results with any treatment. It is considered a poor prognostic factor in head and neck malignancies [11].

As the frequency of cutaneous metastasis varies between 0.7% and 2.4%, a vigilant history and thorough examination are mandatory in such cases. Generally, such cutaneous metastases are found near the primary site, but the appearance of such lesions in other body areas should be examined with keen attention. All such lesions should be viewed with a potential high index of suspicion for malignancy. Tissue biopsy or cytology should be preferably done in cutaneous nodules in view of high suspicion for malignancy or metastasis, even in those with a benign-looking appearance on examination.

## Conclusions

This case report will be an addendum to the already reported cases of cutaneous metastasis from head and neck squamous cell carcinoma. There is no agreed consensus and established guidelines on the management of these kinds of cancers. Treatment remains palliative most of the time, but multimodality management should always be kept as an option depending on a case-to-case basis. This case will increase the knowledge and insight of treating clinicians about the possibility of such a rare expression of oral squamous cell carcinoma in the post-operative period.
